# Codifying healthcare – big data and the issue of misclassification

**DOI:** 10.1186/s12871-015-0165-y

**Published:** 2015-12-15

**Authors:** Karim S. Ladha, Matthias Eikermann

**Affiliations:** Department of Anesthesia, Toronto General Hospital and University of Toronto, Toronto, ON M5G 2C4 Canada; Department of Anesthesia, Critical Care and Pain Medicine, Massachusetts General Hospital, Harvard Medical School, Boston, MA 02115 United States

## Abstract

The rise of electronic medical records has led to a proliferation of large observational studies that examine the perioperative period. In contrast to randomized controlled trials, these studies have the ability to provide quick, cheap and easily obtainable information on a variety of patients and are reflective of everyday clinical practice. However, it is important to note that the data used in these studies are often generated for billing or documentation purposes such as insurance claims or the electronic anesthetic record. The reliance on codes to define diagnoses in these studies may lead to false inferences or conclusions. Researchers should specify the code assignment process and be aware of potential error sources when undertaking studies using secondary data sources. While misclassification may be a short-coming of using large databases, it does not prevent their use in conducting meaningful effectiveness research that has direct consequences on medical decision making.

## Background

The rise of electronic medical records has led to a proliferation of large observational studies that examine the perioperative period. In contrast to randomized controlled trials (RCTs), these studies have the ability to provide quick, cheap and easily obtainable information on a variety of patients and are reflective of everyday clinical practice. Additionally these databases, with their large sample sizes, allow us to study rare but serious conditions such as reintubation that are difficult to detect in RCTs. However, it is important to note that the data used in these studies are often generated for billing or documentation purposes such as insurance claims or the electronic anesthetic record. In other words, it is “found data” or data which is not collected primarily for research. This renders the results of these studies susceptible to issues and biases not faced when dealing with traditional randomized controlled trials.

The study by Thomas et al. recently published in BMC Anesthesiology [1] highlights one of these concerns, that is misclassification or measurement error. In their study, the authors examined trends in the International Classification of Diseases 9^th^ edition (ICD-9) coding of sepsis and compared it to trends in clinically defined sepsis at a single tertiary center. They discovered an increase in the medical coding of sepsis over time that was not accompanied by a concomitant increase in clinically defined sepsis. This work highlights the caution that must be taken when using administrative databases to study disease trends and outcomes but also has several limitations that should be considered when determining its implications.

## Main text

Nosology refers to the discipline of the systematic classification of diseases. While the field has ancient roots, its introduction into Western society was made by Thomas Syndenham during the 17^th^ century [2]. The importance of nosology has continued to increase over time and the field has become particularly relevant as technology continues to play a more prominent role in the delivery of healthcare. ICD-9 codes are perhaps the most commonly used classification scheme in perioperative epidemiologic research. The generation of these codes is undoubtedly susceptible to error at several different points along the path from patient admission to the inclusion into a database [3]. The concern is that if researchers subsequently use these codes that are prone to error in studies, then false conclusions may be made.

It has been suggested that validation studies be routinely performed to understand the accuracy of specific ICD-9 codes before using them in an analysis [4]. This type of study involves the comparison of administrative codes to data abstracted from chart review. The work of Thomas et al. [1], falls short of invalidating codes for sepsis since the authors did not investigate the accuracy of coding but rather looked at their use over time. Thus, it is unclear what is responsible for the discrepancy that they discovered and it could be that coding for sepsis became more accurate over time.

Validation studies are not a panacea for misclassification bias. First, validation studies are usually undertaken at a single center since large national databases are typically de-identified. It is plausible and likely that coding practices differ across institutions as the coders undoubtedly have varying levels of training/experience between centers. Thus the generalizability of validation studies is unclear. The issue becomes murkier when considering diseases that do not have strict diagnostic criteria such as acquired muscle weakness in the intensive care unit [5], which creates variation amongst clinician documentation as well.

There are no set criteria or cut-offs in defining acceptable accuracy of a particular code for use in a study. The validity of a specific code can be described in terms of its specificity, sensitivity, negative predictive value and positive predictive value. Which of these measures is most important can depend on the question that is being asked of the data. Finally some would argue that the level of accuracy is less important than the pattern of error. If there is random or non-differential misclassification than it has been traditionally argued that this would bias estimates towards the null, although this notion has been challenged [6].

## Conclusion

While misclassification is a threat to the validity of a study, it is not a sufficient reason to dismiss observational research using administrative datasets. To do so, would be to lose a major opportunity to gain insights into how to make healthcare delivery more efficient and safer. Rather, misclassification should be viewed as simply a source of potential bias that must be considered when interpreting the results of these studies. Although validation studies may provide insight into the accuracy of some codes, it is neither practical nor possible to perform validity studies on every single ICD-9 code used in a particular investigation. One potential solution is to perform sensitivity analyses to determine how sensitive effect estimates are to misclassification [7].

The practice of evidence-based medicine is the application of the best available knowledge. This entails systematically identifying and evaluating appropriate literature, and integrating it with clinical expertise [8]. The traditional view of the evidence-based pyramid ranks evidence from the top (meta-analyses of well performed RCTs) to the bottom (expert opinion). However, each type of evidence has a unique set of benefits and disadvantages [9]. In practice, there is no perfect defense against misclassification and like any type of study design, repeated investigations of the same question using a variety of databases and analytic techniques is likely the best way to obtain causal inference.

## References

[1] Thomas BS, Jafarzadeh SR, Warren DK, McCormick S, Fraser VJ, Marschall J. Temporal trends in the systemic inflammatory response syndrome, sepsis, and medical coding of sepsis. http://www.biomedcentral.com/1471-2253/15/169 10.1186/s12871-015-0148-z PMC4657245 26597871

[2] Snider GL (2003). Nosology for our day: its application to chronic obstructive pulmonary disease. Am J Respir Crit Care Med.

[3] O'Malley KJ, Cook KF, Price MD, Wildes KR, Hurdle JF, Ashton CM (2005). Measuring diagnoses: ICD code accuracy. Health Serv Res.

[4] Neuman MD (2015). The importance of validation studies in perioperative database research. Anesthesiology.

[5] Farhan H, Moreno-Duarte I, Latronico N, Zafonte R, Eikermann M. Acquired muscle weakness in the surgical intensive care unit: nosology, epidemiology, diagnosis, and prevention. Anesthesiology. 2015;1. 10.1097/ALN.0000000000000874 26445385

[6] Jurek AM, Greenland S, Maldonado G, Church TR (2005). Proper interpretation of non-differential misclassification effects: expectations vs observations. Int J Epidemiol.

[7] Chu H, Wang Z, Cole SR, Greenland S (2006). Sensitivity analysis of misclassification: a graphical and a Bayesian approach. Ann Epidemiol.

[8] Greenhalgh T, Howick J, Maskrey N, Evidence Based Medicine Renaissance Group (2014). Evidence based medicine: a movement in crisis?. BMJ.

[9] Slater AE (2013). Advantages and disadvantages of evidence from clinical trials. Br J Clin Gov.

